# Voltammetric determination of paracetamol in tablet formulation using Fe (III) doped zeolite-graphite composite modified GCE

**DOI:** 10.1016/j.heliyon.2019.e01663

**Published:** 2019-05-10

**Authors:** Meareg Amare

**Affiliations:** Department of Chemistry, Bahir Dar University, Ethiopia

**Keywords:** Pharmaceutical chemistry, Analytical chemistry

## Abstract

Although paracetamol is known to have excellent safety profile at recommended therapeutic doses, health effects are also reported at acute overdoses. A sensitive and selective voltammetric method using Fe(III) encapsulated zeolite/graphite composite modified glassy carbon electrode is presented in this work for the determination of paracetamol in tablet formulations. In contrast to the unmodified electrode, a fourfold increase of cyclic voltammetric oxidative peak current paralleled by reduced potential difference (*ΔE*_*p*_) at the modified electrode confirmed electrocatalytic property of the modifier towards oxidation of paracetamol. The oxidative peak current showed linear dependence on concentration range 0.5–200 μM with *R*^2^ and LOD of 0.9989 and 0.01 μM, respectively. The paracetamol content of four brands of tablet samples was found in the range 95.95 ± 0.23–103 ± 0.52% of the theoretical values. Recovery results between 94.54 ± 0.82 and 102 ± 0.34% for spiked paracetamol in tablet samples validated the selectivity of the method for determination of paracetamol in real samples.

## 1 Introduction

Paracetamol (PCT) is one of the most common oral analgesics and antipyretics used for the relief of fever, headache, menstrual cramps and other minor aches and pains [1], [2]. It is also useful in the management of more severe pains, where it allows lower dosages of additional Non-Steroidal Anti-Inflammatory Drugs (NSAIDs) or opioid analgesics to be used, thereby minimizing overall side-effects [3].

**Scheme 1:**
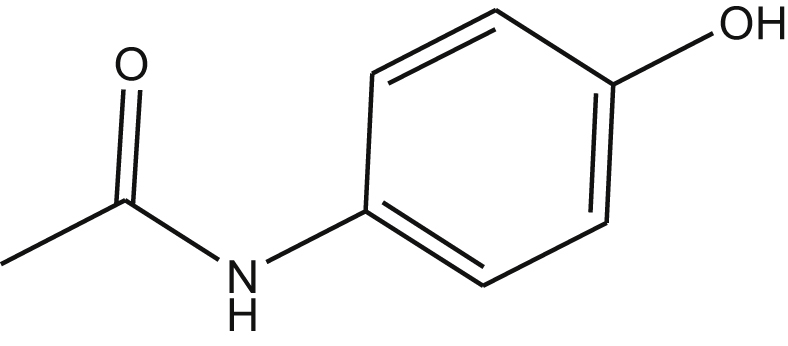
Chemical structure of paracetamol (acetaminophen).

Although PCT (Scheme 1) is renowned to have excellent safety profile at recommended therapeutic doses, acute overdose or misused in at-risk populations is also known to exhibit few side effects including fatal hepatotoxicity, often heightened with use of alcohol [3], [4], [5]. Therefore, there is a clear need to find a sensitive and selective analytical technique that enables to monitor the PCT content of the various sources including pharmaceuticals and body fluids.

High performance liquid chromatography [6], [7], spectrometry [7], [8], [9], [10], and amperometry [11], [12], [13], [14] are among the reported methods for determination of PCT in real samples including pharmaceutical formulations. Although most of them are standard methods due to their high sensitivity and reproducibility, these conventional methods are also known to have some limitations including high instrumental and analysis cost, skilled man power, and most of them are not environmentally-friendly. In contrast to these conventional analytical methods, electroanalytical methods offer remarkable sensitivity, accuracy, and precision in addition to a large dynamic range, with relatively low instrumentation cost [15], [16]. Thus, there is a clear need for development of suitable electroanalytical methods for determination of PCT in various matrices. Voltammetric techniques using modified electrodes have been reported [17], [18], [19], [20], [21], [22], [23], [24], [25] for determination of PCT in pharmaceutical formulations.

Zeolites are crystalline microporous solids that contain many channel-networks proving molecular-sized cages and passageways for excellent steric control of reaction paths which is attributed to the pore geometry [24]. Both the undoped and transition metal-doped zeolite modified electrodes exhibited catalytic properties towards electrochemical determination of numerous analytes [25], [26], [27], [28], [29]. Nevertheless, to the best of our knowledge voltammetric determination of PCT in tablet samples using iron (III) exchanged zeolite/graphite composite modified glassy carbon electrode has not been reported. Thus, we presented the application of Fe(III) doped zeolite-graphite composite modified glassy carbon electrode (FZ-G/GCE) for determination of PCT in tablet formulation.

## 2 Experimental

### 2.1 Chemicals and reagents

Sodium Y Zeolite powder (Merck), anhydrous ferric chloride (99.99%, Merck), graphite powder (Blulux Laboratories (p) Ltd.), polystyrene (Merck), dichloromethane (99.9%, Carlo Erba reagents), tetrahydrofuran (99.5%, Blulux laboratories (p) Ltd), paracetamol (99.8%, Merck), uric acid (99.0%, Labort Fine Chem Pvt Ltd), orthophosphoric acid (85.74%, Fisher Scientific), potassium dihydrogen orthophosphate (99.0%, Titan Biotech Ltd), di-potassium hydrogen orthophosphate (99.0%, Titan Biotech Ltd), potassium nitrate (>99.0%, Merck) are among the chemicals used. All chemicals and reagents were of analytical grade and hence used without prior treatment.

### 2.2 Apparatus

CHI 760d Electrochemical Workstation (Austin, Texas, USA), ultrasonicator (Indiamart), pH meter (Adwa instruments kit), electronic balance (Nimbus, Adam equipment, USA), centrifuge (Thermo Fisher Scientific) are among the apparatus used.

### 2.3 Procedures

#### 2.3.1 Preparation of standard paracetamol solutions

100 mL of 10 mM standard paracetamol stock solution was prepared in a 0.1 M PBS (pH 7.0) from which 1 mM in pH 7.0 as intermediate and working solutions of various concentration (0.5, 1, 5, 10, 20, 40, 60, 80, 100, 150, and 200 μM in pH 4.5 PBS) were prepared through serial dilution.

#### 2.3.2 Preparation of Fe^3+^ doped zeolite-graphite composite modified GCE

First, iron (III) doped zeolite was prepared following minor modification of previously reported procedure [30]. Briefly, 1 g of sodium Y zeolite was lightly ground and placed in 250 mL of 0.01 M FeCl_3_ solution and stirred for 48 h. The Fe^3+^ doped zeolite was then collected by decantation, carefully washed with HCl solution (pH 2.0) to remove occluded material and surface-adherent salt, washed with distilled water to remove chloride ion, and finally dried at room temperature.

To prepare the iron (III) doped zeolite/graphite composite modified GCE, ground mixture of 50 mg of the iron (III) doped zeolite and an equal amount of graphite powder was put in to an eppendorf tube to which 10 mg of polystyrene, 0.25 mL tetrahydrofuran, and 0.35 mL of dichloromethane were added sequentially. To facilitate the dispersion of the composite, the solution was sonicated for 5 min. Finally, 10 μL of the mixture was casted on the surface of a mirror-like polished GCE and left on air for at least 30 minutes before use.

#### 2.3.3 Tablet sample preparation

Paracetamol tablets of four brands; Adol Julphar (Ethiopia), Panadol advance (Kenya), Kelvin (India), and Para-Denk (Germany) all labeled 500 mg PCT/tablet were purchased from a pharmacy in Bahir Dar city for analysis of their PCT content using the developed method. Five weighed tablets from each brand were powdered using mortar and pastel and homogenized. 100 mL stock solution of PCT tablet was prepared by dissolving 0.1 g of the powder in pH 4.5 PBS. Furthermore, 100 mL working tablet solution was prepared by dissolving 1.0 mL of the tablet stock solution in pH 4.5 PBS and kept in a refrigerator for its PCTcontent analysis.

To further validate the applicability of the developed method for determination of PCT in real samples like tablet formulations, recovery and interference studies were conducted. For method validation using recovery and interference tests, four Ethiopian brand tablet sample solutions designated as unspiked (A), spiked with 50 μM PCT (B), spiked with 50 μM PCT and 40 μM uric acid (C), and spiked with 50 μM PCT and 80 μM uric acid (D) were prepared in pH 4.5 PBS. While results for solutions A and B was used for recovery study, results for solutions B-D was used for interference study.

#### 2.3.4 Electrochemical measurements

A conventional three-electrode system was employed with a bare GCE (3 mm in diameter, CH Instruments, Inc), or iron (III) doped zeolite/graphite composite modified glassy carbon electrode (FZGC/GCE) as working electrode, silver/silver chloride (Ag/AgCl) as reference electrode and a platinum coil as a counter electrode.

While cyclic voltammetry was used to investigate the electrochemical behavior of PCT at the composite modified and unmodified glassy carbon electrodes, effect of scan rate on oxidative peak current, and dependence of both the oxidative peak current and peak potential of PCT at the surface of the composite modified glassy carbon electrode, square-wave voltammetry under default parameters (amplitude 25 mV, step potential 4 mV, and frequency 25 Hz) was employed for the quantitative analyses of PCT in PCT tablet formulations of different brands.

## 3 Results and discussion

### 3.1 Electrochemical behavior of paracetamol at FZ-G/GCE

**Fig. 1:**
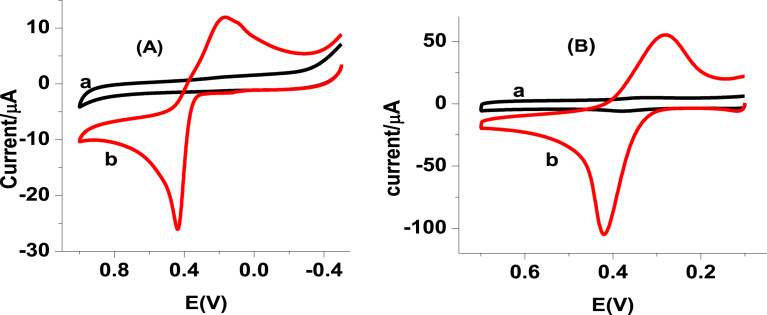
Cyclic voltammograms of (A) UGCE, and (B) FZ-G/GCE in 0.1 M pH 7.0 PBS containing (a) no and (b) 1 mM PCT. Scan rate 100 mV s^-1^.

Although with different peak potential difference and peak current intensity, oxidative and reductive peaks appeared in opposite scan directions at both electrodes; unmodified glassy carbon electrode (UGCE) and iron (III) doped zeolite-graphite composite modified glassy carbon electrode (FZ-G/GCE) depicted in Fig. 1. The peak potential difference and peak current of 1 mM PCT at the two electrodes are summarized in Table 1. In contrast to the UGCE, an improved peak potential difference and hence improved reversibility and fivefold anodic current enhancement at the modified electrode confirmed the modification of the electrode surface with a material that possesses electrocatalytic activity towards the reaction of PCT which might be accounted for an improved surface area as a result of surface modification. Large potential difference between the cathodic and anodic peaks supplemented by a peak current ratio larger than unity indicated the irreversibility of the reaction of PCT even at the modified electrode.

**Table 1:** Summary of peak potentials and currents of 1 mM PCT at UGCE and FZ-G/GCE.

Electrode	E_pa_ (mV)	E_pc_ (mV)	ΔE_p_ (mV)	I_pa_ (μA)	I_pc_ (μA)
GCE	437	164	273	26	12
FZ-G/GCE	421	279	142	105	55

### 3.2 Effect of scan rate on the oxidative peak current of paracetamol at FZ-G/GCE

**Fig. 2:**
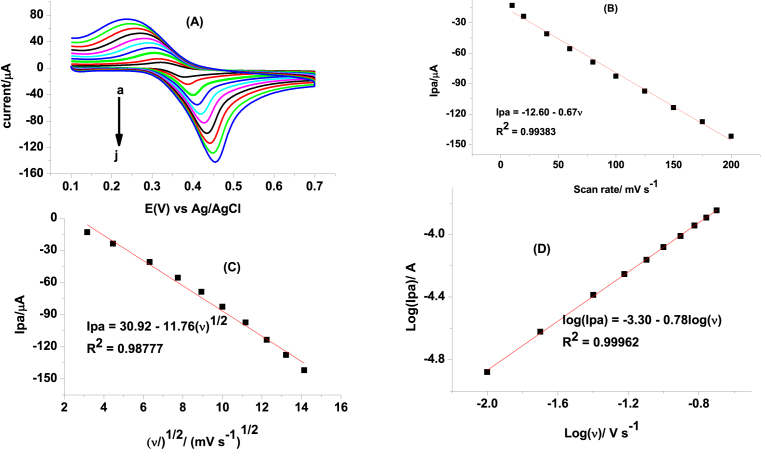
(A) Cyclic voltammograms of FZ-G/GCE in pH 7 PBS containing 1 mM PCT at various scan rates (a–j: 10, 20, 40, 60, 80, 100, 125, 150, 175, and 200 mV s-1, respectively), (B) plot of oxidative peak current versus scan rate, (C) plot of oxidative peak current versus square root of scan rate, and (d) plot of log of oxidative peak current versus log of scan rate.

**Fig. 3:**
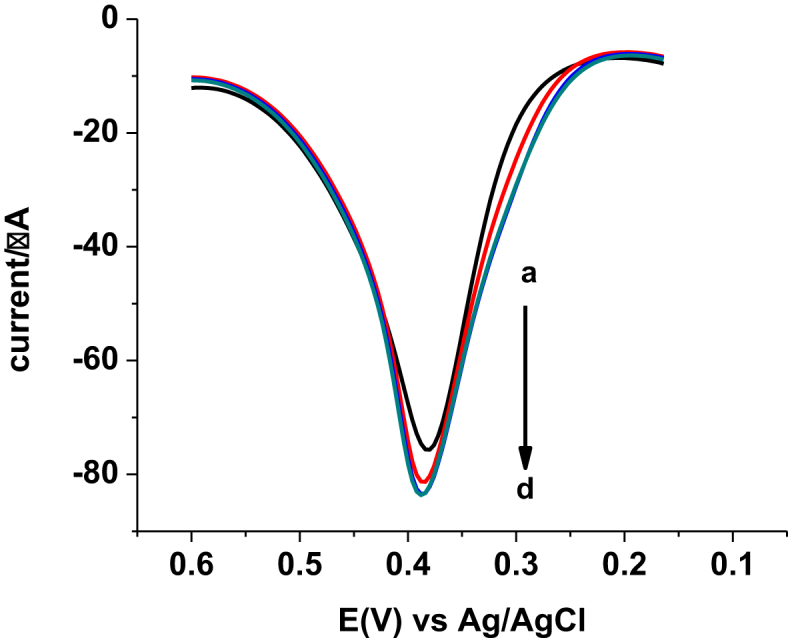
Linear sweep voltammograms of FZ-G/GCE in pH 7 PBS containing 1 mM PCT at different quit times (a–d: 0, 10, 20, and 30 s, respectively).

In order to investigate the reversibility of PCT and rate determining step during its reaction at the composite modified electrode, the effect of scan rate on the peak potential and peak current was studied. The observed peak potential shift with increasing scan rate (Fig. 2A) confirmed the irreversibility of the reaction. Moreover, comparable determination coefficients for the dependence of peak current on the scan rate (Fig. 2B) and on the square root of scan rate ((Fig. 2C) indicated that the reaction was influenced by both the adsorption of the analyte on the surface of the modified electrode and diffusion mode of mass transport. This was also supported by the slope value 0.78 of plot of log of oxidative peak current versus log of scan rate which is above 0.5 for diffusion and 1.0 for adsorption [31]. To evaluate the extent adsorption influences the kinetics of the oxidation reaction of PCT, the current response of the electrode was monitored as a function of time exposure to 1 mM PCT in pH 7 PBS (Fig. 3).

As can be seen from the figure, the electrode revealed almost comparable oxidative current irrespective of the time of exposure of the electrode surface for PCT confirming that the reaction was more influenced by diffusion.

### 3.3 Effect of the type of supporting electrolyte on the redox property of PCT at FZ-G/GCE

**Fig. 4:**
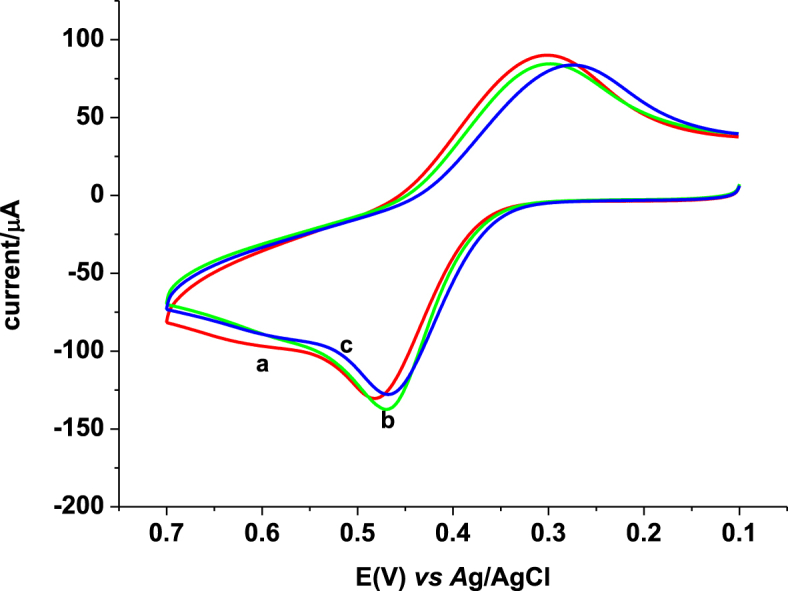
Dependence of the electrochemical behavior of PCT at FZ-G/GCE on pH 5.0 of the type of supporting electrolyte used (a–c: ABS, PBS, and RBS, respectively).

The electrochemical response of FZ-G/GCE for PCT in different types of buffers was investigated. Due to the fact that acetate buffer (ABS), phosphate buffer (PBS), and Britton-Robinson buffer solutions (BRS) exhibit common buffering capacity at pH 5, the three buffers all of pH 5 were used for our context. Fig. 4 presents the voltammograms of 1 mM PCT in pH 5 of the three buffer solutions. As can be observed from the figure, all of the supporting electrolytes revealed comparable oxidative peak current although the PBS looked the best. Thus for its better oxidative peak current value and wider buffering capacity, the PBS was chosen as the supporting electrolyte in this work.

### 3.4 Effect of pH of PBS on the peak current and peak potential of PCT at FZ-G/GCE

**Fig. 5:**
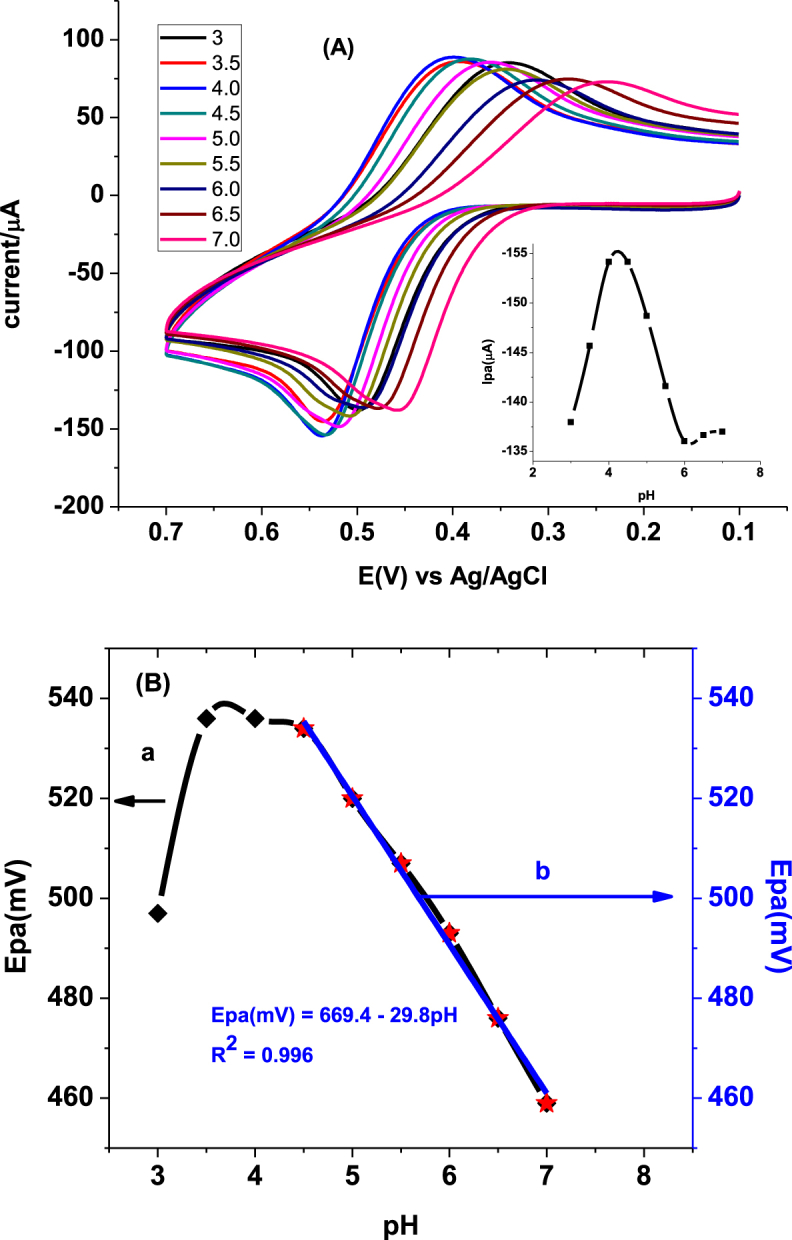
(A) Cyclic voltammograms of FZ-G/GCE in PBS of various pHs (3.0, 3.5, 4.0, 4.5, 5.0, 5.5, 6.0, 6.5, 7.0, respectively) containing 1.0 mM PCT, and (B) plot of oxidative peak potential as a function of pH (a) in the entire range and (b) in the pH range (4.5–7.0) where it showed linear change.

As depicted in Fig. 5A, the oxidative peak current showed three trends; an increasing trend from pH 3.0–4.0, constant current between 4.0 and 4.5, and then decreasing trend beyond pH 4.5 (Inset of Fig. 5A). In our case, pH 4.5 was taken as the optimum pH value.

The dependence of the peak potential on the pH of the buffer solution was also investigated. As witnessed from Fig. 5B (curve a), the oxidative peak potential varied with the pH in the entire range of pH but still following different trends. While the potential shifted in the negative direction from pH 3.0 to 4.0, a shift in the opposite direction was observed for pH values beyond 4.5. The observed peak potential shift indicated participation of protons during the oxidation of PCT at the surface of the modified electrode. Moreover, the potential shift with pH in the range 4.5–7.0 showed linear dependence with determination coefficient (*R*^2^) and slope of 0.996 and 29.8 mV, respectively.

### 3.5 FZ-G/GCE for the determination of PCT

#### 3.5.1 Square-wave voltammetric behavior of PCT at the modified electrode

**Fig. 6:**
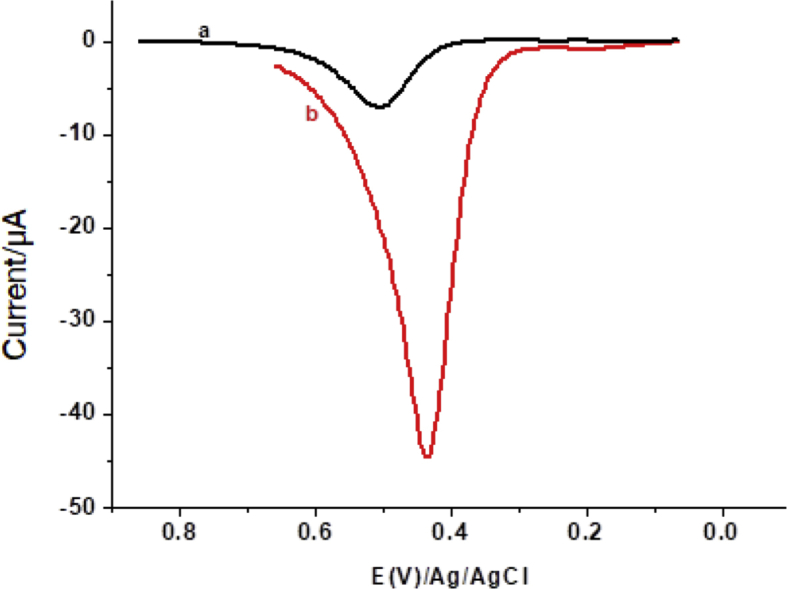
Corrected for blank square-wave voltammograms of 1 mM PCT in pH 4.5 PBS at (a) unmodified glassy carbon electrode and (b) FZGCE. SWV amplitude, step potential, and frequency of 25 mV, 4 mV, and 15 Hz, respectively.

Due to its sensitivity, square-wave voltammetry (SWV) was selected for determination of PCT. Fig. 6 depicts background corrected square-wave voltammograms of 1 mM PCT in pH 4.5 PBS at the unmodified (curve a) and modified (curve b) electrodes. In contrast to the UGCE (curve a), an oxidative peak with sixfold enhanced peak current and reduced overpotential at the FZ-G/GCE (curve b) confirmed the electrocatalytic role of the modifier towards oxidation of PCT. This confirmed the applicability of the composite modified glassy carbon electrode for determination of PCT.

#### 3.5.2 Dependence of the oxidative current on the concentration of PCT

**Fig. 7:**
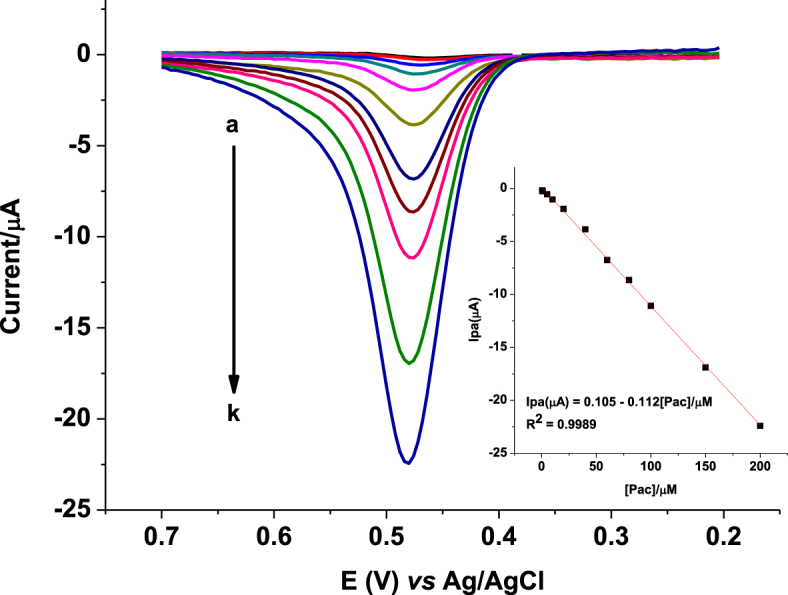
Background corrected square-wave voltammograms of FZ-G/GCE in pH 4.5 PBS containing various concentrations of PCT (a–k: 0.5, 1, 5, 10, 20, 40, 60, 80, 100, 150, and 200 μM, respectively). Inset: plot of oxidative peak current versus concentration of PCT.

Fig. 7 presents the back ground corrected square-wave voltammograms of various concentrations of PCT in pH 4.5 PBS at the composite modified GCE. The oxidative peak current showed linear dependence on the PCT concentration (Inset of Fig. 7) in the range 0.5–200 μM with determination coefficient (*R*^2^) and detection limit (3SD_blank_ for *n* = 5) of 0.9989 and 0.01 μM, respectively validating the applicability of the method for trace level determination of PCT.

#### 3.5.3 Determination of PCT content in paracetamol tablet formulations

**Fig. 8:**
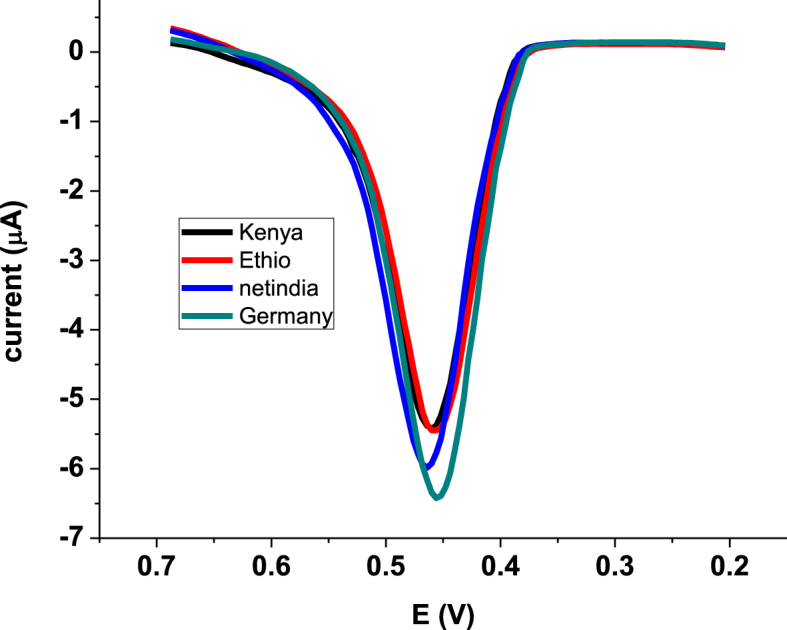
SWVs of FZ-G/GCE in pH 4.5 PBS containing PCT tablet samples of different brands (Ethiopia, India, Germany, and Kenya).

The developed method was used for determination of PCT content in four brands tablet samples prepared as described under the experimental part. While Fig. 8 presents the square-wave voltammograms for the studied four tablet brands, the detected PCT content in each brand tablet and hence the percent detected relative to the theoretical label and corresponding tablet mass are summarized in Table 2.

**Table 2:** Summary of the detected PCT content of the analyzed tablet brands labeled as 500 mg/tablet.

Tablet brand	Mass of tablet (mg/tablet)^a^	Nominal PCT content (mg/100 mg tablet)	PCT per 100 mg tablet sample (mg)^b^	Found PCT in percent^c^
Adol Julphar (Ethiopia)	646.88	77.29	74.86±0.25	96.85±0.25
Para-Denk (Germany)	594.22	84.14	87.31±0.50	103.76±0.50
Kelvin (India)	584.48	85.55	82.08±0.23	95.95±0.24
Panadol advance (Kenya)	671.54	74.46	73.53±0.98	98.76±0.99

a Mean mass of tablet (*n* = 5).

b Detected mean PCT ±%RSD.

c ±%RSD for triplicate measurements.

Results illustrating PCT content in the range 95.95–103.76% of what is expected with %RSD value below 1% showed the accuracy and precision of the developed method which further validated the applicability of the method for determination of PCT in a complex matrix.

#### 3.5.4 Recovery and interference study

**Fig. 9:**
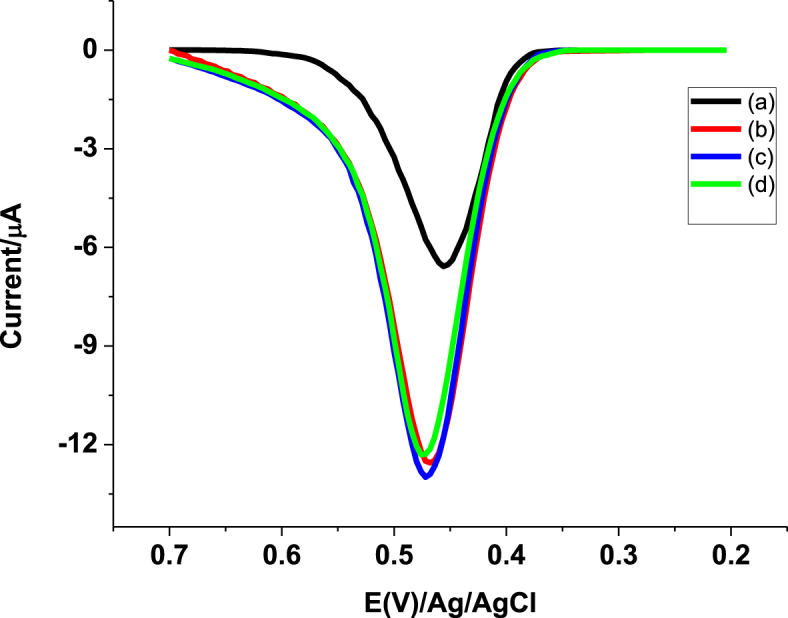
SWVs of pH 4.5 PBS containing (a) Ethiopian PCT tablet solution, (b) a + 55 μM standard PCT, (c) b 40 μM UA, and (d) b + 80 μM UA.

To further validate the developed method, recovery of spiked standard PCT in the absence and presence of a potential interferent uric acid (UA) was investigated. Four Ethiopian brand tablet sample solutions were prepared each containing 0.22 mg tablet powder in 25 mL pH 4.5 PBS. The resulting solutions designated as a-d were then spiked with standard PCT and uric acid solutions of 0.00 & 0.00, 55.00 & 0.00, 55.00 & 40.00, and 55.00 & 80.00 μM, respectively. As can be seen from Fig. 9, voltammograms with similar peak currents were recorded for the spiked PCT sample both in the absence and presence of uric acid conforming the selectivity of the developed method. Recovery results of the spiked PCT both in the absence and presence of uric acid (Table 3) in the range of 94.54–102.03 still with low percent relative standard deviation validated the applicability of the developed method for determination of PCT in tablet formulations irrespective of the amount of the potential interferent, UA.

**Table 3:** Summary of recovery results of 55.00 μM PCT from tablet solutions containing 0.22 mg Ethiopian brand both in the absence and presence of 40 and 80 μM UA.

Sample	Initially present PCT (μM)	Added PCT (μM)	Added UA (μM)	Found PCT (μM)^a^ (mean±SD)	%Recovery^a^
(a)	60.00	0.00	0.00	59.81±0.43	====
(b)	60.00	55.00	0.00	114.54±0.61	99.16±0.54
(c)	60.00	55.00	40.00	116.12±0.38	102.03±0.33
(d)	60.00	55.00	80.00	112.76±0.74	94.54±0.87

a Mean ± %RSD for n = 3.

#### 3.5.5 Performance of the developed method compared to reported works

The performance of the developed electrode in this work was compared with selected previously reported electrodes in terms of the linear range, limit of detection, nature of the substrate and cost of material used for modification. As can be seen from Table 4, the present electrode showed the list limit of detection except the electrode modified with palladium which is has toxic effect on human health. Therefore, the reported method using the most available glassy carbon electrode as a substrate and a cheap zeolite modifier showed a comparable performance even with the methods that have used expensive otherwise toxic electrode modifiers.

**Table 4:** Performance of the developed method in contrast to selected reported works.

Substrate	Modifier	Method	Dynamic range	LOD in μM	Ref
GCE	MWCNT/TiO_2_	CV	10–120 μM	11.77 μM	[17]
CPE	MWCNT/platinum nanoparticles	AdSDPV	0.351–56.1 μM	0.0279 μM	[19]
GO	Palladium	DPV	0.005–0.5 μM	0.0022 μM	[20]
GCE	4-aminobenzene sulfonic acid	DPV	0.6–9 μM	0.0933 μM	[22]
CPE	Co(II) modified zeolite	CV	0.1–190 μM	0.04 μM	[25]
GCE	Fe(III) doped zeolite/graphite composite	SWV	0.5–200 μM	0.01 μM	This study

## 4 Conclusion

Cyclic voltammetry was employed for the study of the electrochemical behavior of PCT, dependence of peak current on the pH of the solution and scan rate. In contrast to the unmodified glassy carbon electrode, Iron (III) exchanged zeolite-graphite composite modified glassy carbon electrode showed catalytic property towards oxidation of PCT. A square-wave voltammetric method using the FZ-G/GCE was used for determination of PCT even in a tablet formulation with a complex matrix. Wide dynamic concentration range, low detection limit, excellent recovery results and hence accuracy, high recovery results even in the presence of a potential interferent and hence its selectivity, and extremely low percent relative standard deviation values demonstrating its precision validated the applicability of the developed method for determination of PCT in tablet samples. The PCT content of the studied tablet samples ranged between 95.95 to 103.76% of their labels confirming the efficiency of the developed method.

## Declarations

### Author contribution statement

Meareg Amare: Conceived and designed the experiments; Performed the experiments; Analyzed and interpreted the data; Contributed reagents, materials, analysis tools or data; Wrote the paper.

### Funding statement

This research did not receive any specific grant from funding agencies in the public, commercial, or not-for-profit sectors.

### Competing interest statement

The authors declare no conflict of interest.

### Additional information

No additional information is available for this paper.
